# Identification of GPI-Anchored Wall Transfer Protein 1 Modulators for Fungal Infections Through Generative AI and Physics-Based Approaches

**DOI:** 10.3390/ijms27114767

**Published:** 2026-05-25

**Authors:** Ibrahim A. Alsarra, Rupesh Chikhale, Abdullah M. Al-Mohizea, Md Ataul Islam

**Affiliations:** 1 Department of Pharmaceutics, College of Pharmacy, King Saud University, P.O. Box 2457, Riyadh 11451, Saudi Arabia; ialsarra@ksu.edu.sa (I.A.A.); amohizea@ksu.edu.sa (A.M.A.-M.); 2 Department of Pharmaceutical and Biological Chemistry, School of Pharmacy, University College London, London WC1N 1AX, UK; r.chikhale@ucl.ac.uk; 3 SilicoScientia Private Limited, Nagananda Commercial Complex, No. 07/3, 15/1, 18th Main Road, Jayanagar 9th Block, Bengaluru 560041, India; 4 SilicoScientia Private Limited, Centre for Cellular and Molecular Platforms (C-CAMP), GKVK Campus, Bellary Road, Bengaluru 560065, India

**Keywords:** GPI-anchored wall transfer protein 1, fungal infections, generative artificial intelligence, machine learning, SilicoXplore, virtual screening

## Abstract

Glycosylphosphatidylinositol (GPI) anchored wall transfer protein 1 (GWT1), a fungal-specific inositol acyltransferase, catalyzes the palmitoylation of GlcN-PI in GPI-anchor biosynthesis, crucial for mannoprotein trafficking and attachment, which are vital for cell wall integrity, biofilm formation, and virulence. More than 60,000 AI-generated molecules produced using REINVENT4 were screened using ADMET-AI and GNINA. DeepSA and PharmacoNet were used to select synthesizable and pharmacophorically rich molecules. The dynamic behaviour was explored using molecular dynamics (MD). Finally, molecular reactivity was assessed using density functional theory (DFT). After ADMET filtering, 6190 compounds were docked against GWT1, of which 315 showed better predicted binding energies than the co-crystal ligand. DeepSA identified 105 readily synthesizable candidates, and PharmacoNet retained 32 compounds with favourable pharmacophoric features, from which four final candidates (AF_M1, AF_M2, AF_M3, and AF_M4) were prioritized for further analysis. MD simulation suggested stable binding behavior towards GWT1. DFT analysis indicated favourable electronic properties, low HOMO-LUMO energy gaps, and stable optimized geometries. These molecules could serve as promising lead candidates and potential new therapeutic agents for invasive fungal infections, pending validation.

## 1. Introduction

Fungal infections are a major global health issue, including a wide range of diseases from superficial and skin infections to deeper, mucosal, and potentially life-threatening systemic infections, which differ greatly in severity and clinical effects [1]. Each year, they cause an estimated 6.5 million cases of invasive disease and claim approximately 3.8 million lives, of which about 2.5 million are directly attributable to the infection itself [2]. These alarming numbers exceed those of several well-known infectious diseases, including malaria and tuberculosis in some settings, yet fungal diseases remain notably underrecognized and underfunded [3]. The mortality rate caused by key pathogens, such as invasive aspergillosis, affects over 2.1 million people annually, mainly those with chronic obstructive pulmonary disease, cancer, or critical illness, and has a very high crude mortality rate of about 85%, resulting in roughly 1.8 million deaths each year [2]. These primarily impact vulnerable groups like immunocompromised individuals, transplant recipients, cancer patients, and people in intensive care. The crisis worsens due to very limited treatment options, with only a few classes of antifungal drugs currently available, and the rapid emergence of resistance [4,5]. Widespread overuse in both medicine and agriculture has led to the emergence of multidrug-resistant strains, especially *Candida auris*, which often exhibits resistance to multiple first-line treatments and spreads readily in healthcare settings, making control much harder [6]. Diagnosis is frequently delayed or unavailable in resource-poor areas, leading to worse outcomes, longer hospital stays, and higher healthcare costs. As populations with risk factors continue to grow due to aging societies, increasing chronic illnesses, more widespread use of immunosuppressive therapies, and environmental changes, the danger from fungal infections is expected to increase further [7]. In addition to the above, current antifungal options are limited, associated with high toxicity, increasing resistance, and substantial costs, thereby representing a significant burden on public health systems [3]. It is also important to note that for nearly two decades, no class of antifungal drugs has been approved by the US Food and Drug Administration (FDA) [8]. In this context, developing more effective and safer antifungals is an urgent priority [9,10]. The glycosylphosphatidylinositol (GPI) biosynthesis pathway is an essential cellular process for many eukaryotes and has emerged as an attractive target for novel antifungals [11,12]. This pathway is required to anchor mannoproteins to the fungal cell wall. These mannoproteins have critical functions, including providing structural integrity to the cell wall, facilitating adhesion of pathogenic fungi to mucosal surfaces, and promoting their replication at mucosal surfaces, which can result in disseminated infection [13]. The fungal enzyme GWT1 (GPI-anchored wall transfer protein 1), an inositol acyltransferase involved in GPI anchor biosynthesis, is a highly attractive target for the discovery of novel antifungal drugs [14]. GWT1 facilitates the third step of GPI anchor maturation by attaching a palmitoyl group to glucosaminyl-phosphatidylinositol (GlcN-PI) through acylation [15]. This modification is essential for the proper trafficking, membrane attachment, and incorporation of GPI-anchored mannoproteins into the cell wall. These proteins are essential for maintaining fungal cell wall integrity, adhesion, biofilm formation, and virulence. Inhibition of GWT1 causes severe cell wall defects, endoplasmic reticulum stress, and greatly reduces fungal growth and pathogenicity in major human pathogens, including *Candida* spp., *Aspergillus* spp., and *Cryptococcus* spp. [14,16]. Due to its limited sequence homology to the human ortholog PIG-W and its crucial role in major pathogenic fungi, targeting GWT1 with drugs offers a selective mechanism of action distinct from current antifungal classes [17]. The first-in-class inhibitor manogepix (also known as E1210 or APX001A), which is currently progressing through Phase 3 clinical trials, competitively binds within the palmitoyl-CoA pocket of GWT1, as revealed by recent high-resolution cryo-EM structures [18]. This target validation and the demonstrated broad-spectrum activity against yeasts and molds, including multidrug-resistant strains such as *Candida auris*, highlight GWT1 as a promising target for the development of next-generation antifungal agents [19].

Ibrexafungerp and rezafungin were approved by the FDA in 2021 and 2023, respectively, as antifungal agents, but both remain limited in scope. Ibrexafungerp, an oral triterpenoid β-(1,3)-D-glucan synthase inhibitor, is currently indicated only for vulvovaginal and recurrent vulvovaginal candidiasis, while trials for invasive indications are ongoing [20]. Rezafungin, a next-generation echinocandin, targets the same β-(1,3)-D-glucan synthase as older echinocandins and therefore remains vulnerable to FKS-mediated cross-resistance and offers limited mold coverage [21]. Olorofim (F901318), the first orotomide, selectively inhibits fungal dihydroorotate dehydrogenase (DHODH). It entered the Phase 3 OASIS trial (NCT05101187) in 2022 but is not yet approved [22]. Critically, olorofim lacks activity against yeasts and the Mucorales, and single-point DHODH mutations readily confer resistance [23]. The GWT1 inhibitor manogepix is being clinically advanced as the prodrug fosmanogepix, which is rapidly converted to the active moiety by systemic phosphatases [24]. A Phase 2 trial reported 80% treatment success in non-neutropenic candidemia [25], a separate Phase 2 study evaluated fosmanogepix against *Candida auris* infections [25], and the global Phase 3 FAST-IC trial versus caspofungin was initiated in 2026. Limitations persist, however, including poor activity against *C. krusei* and spontaneous GWT1 mutations that confer reduced susceptibility [26].

Due to the high time, resource, and cost requirements, as well as the technical complexity of traditional drug discovery pipelines, physics-based computational methods such as molecular docking, molecular dynamics (MD) simulations, and binding free energy calculations have become a powerful and cost-effective way to speed up virtual screening and lead optimization [27,28,29,30,31]. Moreover, integrating machine learning (ML) techniques has transformed the landscape of drug discovery by enabling rapid exploration of large chemical spaces, precise prediction of ligand affinity, selectivity, and ADMET (adsorption, distribution, metabolism, excretion, and toxicity) properties, and the creation of new scaffolds [32,33,34,35]. This accelerates the discovery of promising, selective molecules while greatly shortening timelines and experimental effort. The efficiency of physics-based and ML approaches is applicable to a broader range of diseases, including the identification of antifungal molecules. Islam et al. considered more than 50 phytocompounds, used molecular docking, ADMET, and MD simulations, and finally proposed three potential compounds for fungal infection in ginger [36]. In a study by Wang et al., the diffusion-based generative platform was used to design antifungal molecules against five targets, and approximately 50% of the molecules exhibited measurable in vitro activity [37]. In a study, a series of phytomolecules was screened using molecular docking, ADMET, and MD simulations, and the top three molecules were identified as crucial for the treatment of fungal infections [38]. Several other studies have successfully applied computational drug discovery and ML methodologies to find promising molecules for fungal infections [38,39,40,41,42,43,44,45].

Accordingly, the primary objective of the present study was to discover potential lead-like inhibitors of GWT1 through an integrated, multi-stage in silico workflow. This encompassed ML-driven *de novo* molecular design to generate structurally diverse and novel scaffolds, structure-based molecular docking for high-throughput prediction of binding poses and affinities, rigorous ADMET profiling to prioritize compounds with favourable drug-likeness, pharmacokinetic properties, and low toxicity risk, physics-based MD simulations to evaluate binding stability, conformational dynamics, and free-energy landscapes, and density functional theory (DFT) calculations to provide quantum-level insights into electronic structure, reactivity descriptors, and the molecular basis of ligand–receptor interactions. This workflow led to the prioritization of four promising GWT1-directed candidate molecules for further investigation.

## 2. Results

**Figure 1:**
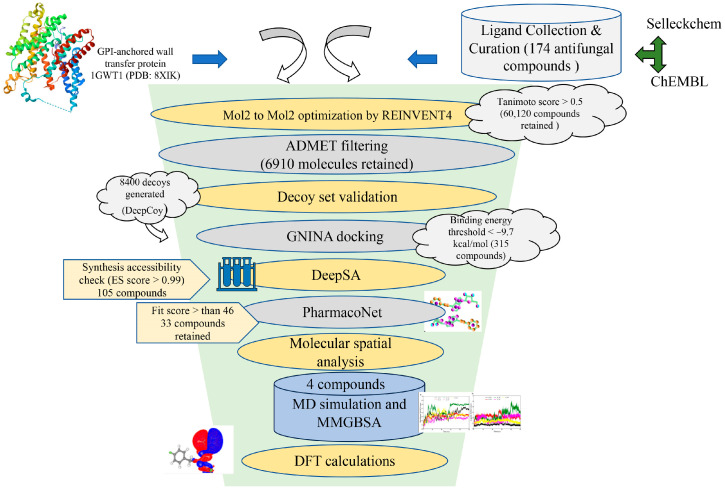
Workflow for the identification of novel GWT1 inhibitors.

A comprehensive computational approach was used to design and identify promising GWT1 inhibitors for antifungal therapeutics. A stepwise workflow of the entire process is shown in Figure 1. Moreover, comprehensive information on stepwise screening molecules for GWT1 is given in Supplementary Data (Table_S1.pdf).

### 2.1. Molecular Library Generation and Screening

#### 2.1.1. Compound Library Generation Using REINVENT4 and Curation

The set of 174 antifungal molecules collected from the Selleckchem [46] and ChEMBL [47] databases was converted to Simplified Molecular Input Line Entry System (SMILES) format using OpenBabel [48]. Subsequently, REINVENT4, a versatile molecular design tool, was used to perform molecular optimization and generate compound libraries. Of the four REINVENT4 modules, Mol2Mol was used to generate the molecular library. This particular model enabled the generation of molecules similar to the parent anti-fungal molecules while retaining the same scaffold responsible for their functional activity. The SMILES representations of 174 antifungal molecules were used as input to incorporate a beam-search decoding strategy and a sampling run mode, generating 148,760 molecules with a specified degree of resemblance to the parent compounds. Furthermore, the compounds were screened for duplicate removal and structural similarity, retaining 111,655 compounds. The SMILES notation for 111,655 molecules with a Tanimoto score is provided in the Supplementary Data (De_novo_designed_molecule.csv). To select highly similar molecules to the input active molecule, molecules with a Tanimoto score of 0.5 or higher were considered. Based on the above criteria, a total of 60,120 molecules were retained for further investigation.

The heatmap of the 60,120 molecules for the Tanimoto coefficient was plotted and is provided in the Supplementary File (Heatmap.jpg). The heatmap showed the structural similarity of 60,120 molecules to A1LVI, using a Tanimoto coefficient of 0.5 or higher. To facilitate visualization, these filtered similarity values were arranged in a compact two-dimensional grid, with each pixel representing one molecule and the axes labelled by molecule index. The colour scale ranges from dark blue (Tanimoto similarity of 0.5, indicating moderate structural overlap) to bright yellow (Tanimoto similarity of 1.0, indicating identical or nearly identical structures). The plot was dominated by dark blue and purple tones, indicating that most selected molecules were moderately similar to the reference. Scattered orange and yellow pixels highlight the smaller number of close structural analogues, while occasional darker horizontal bands revealed groups of compounds with similarity values near the 0.5 threshold. Therefore, this filtered heatmap provided a focused view of the most chemically relevant portion of the library and aids in the rapid identification of promising molecules.

#### 2.1.2. Pharmacokinetics Assessment of the De Novo Generated Compounds

Molecules retained from the Mol2Mol optimization were then subjected to ADMET estimation to assess their pharmacokinetic profiles and toxicity. Different ADMET parameters for 60,120 molecules mentioned above are provided in the Supplementary Data (ADMET_Antifungal_part_#1.zip and ADMET_Antifungal_part_#2.zip). On successful prediction of the ADMET parameters, specific thresholds were applied to various parameters to narrow the chemical space. Molecules exhibiting molecular weight ≤ 500, LogP ≤ 5, HBA < 10, HBD ≤ 5, QED > 0.5, TPSA ≤ 140, AMES ≤ 0.4, Bioavailability ≥ 0.7, Carcinogenicity ≤ 0.5, DILI ≤ 0.5, and HIA ≥ 0.9 (High absorption and can be considered a strong candidate for oral formulation), were retained. By applying the above criteria, 6190 compounds were retained for further analysis.

#### 2.1.3. Molecular Docking and Docking Protocol Validation

##### Validation of Docking Protocol

**Figure 2:**
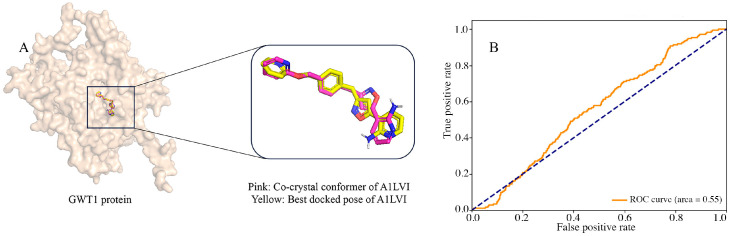
Validation of docking protocol. (**A**) self-docking approach; (**B**) Decoy set validation.

The molecular docking protocol was validated using self-docking and decoy-set approaches, demonstrating that the selected docking parameters could generate conformations of new molecules comparable to the crystallographic conformation. The co-crystal ligand was removed, and the same was re-drawn and iteratively docked at the same position of the GWT1 protein where it was bound. The docking was executed, and the root-mean-square deviation (RMSD) between the best-docked pose and the co-crystal conformation was calculated for each iteration. The best docking protocol was found to be with an RMSD of 1.86 Å and a binding energy of −9.70 kcal/mol. The redocked co-crystal structure was superimposed on the original crystallized conformation, as shown in Figure 2A. The superimposed RMSD of less than 2 Å indicated that the docked conformation might be similar to the crystalized conformation of the same molecule. Further, the decoy-set validation was performed in which 174 active antifungal compounds were considered, and a total of 8400 decoy compounds were generated using the DeepCoy tool [49]. The amalgamated molecules of the active and decoy compounds were docked into the active site of GWT1 using the self-docking-validated protocol. Several statistical parameters, such as accuracy, area under the curve (AUC) of the receiver operator characteristics (ROC), and specificity, were calculated from the confusion matrix. The statistical parameters were an accuracy of 0.588, a ROC-AUC of 0.553, and a specificity of 0.870. The ROC-AUC plot for the docking protocol validation is given in Figure 2B. The self-docking results supported acceptable pose reproduction, while decoy-set validation showed modest enrichment performance, indicating that docking should be interpreted as one prioritization layer rather than a standalone predictor of activity. Hence, self-docking and decoy set validations indicated that the best-docked pose might be similar to the crystallized conformer, and that active molecules might show moderately higher binding affinity than inactive molecules.

##### Molecular Docking

**Figure 3:**
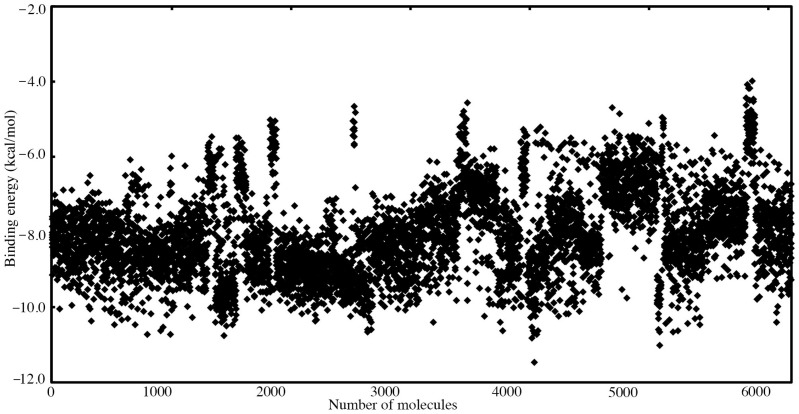
The binding energy of 6190 ADMET-filtered molecules.

To further screen 6190 ADMET-filtered candidates, molecular docking was performed against the GWT1 target to assess their potential binding affinity and pose within the active site. The prepared GWT1 receptor and the ligand library in SDF format were given as inputs for the docking simulations. For the active site, the A1LVI was considered the reference molecule. Docking results were analyzed by evaluating both the estimated binding free energies (in kcal/mol) and the detailed binding orientations of each compound relative to key residues in the GWT1 active site. The binding energy of 6190 molecules is given in Figure 3. Also, the SMILES and binding energies of 6190 molecules are provided in the Supplementary Data (Docking_binding_energy.csv). Hereafter, the molecules are numbered A1 to A6190. It is important to note that all ADMET-filtered molecules yielded docked poses with predicted binding energies ranging from −3.97 to −11.47 kcal/mol.

A key selection criterion was set using the binding energy of the reference ligand A1LVI (−9.70 kcal/mol) as the threshold. This value served as a benchmark reflecting experimentally informed binding strength, ensuring that only compounds predicted to engage the target more favorably through stronger energetic interactions, better shape complementarity, or improved key contacts. Compounds with binding energies more negative than −9.70 kcal/mol, exceeding this threshold, were considered to have superior predicted affinity. After this comparison, 315 molecules were identified as having better docking scores than A1LVI. These high-affinity hits were considered for further screening. The binding energy and SMILES are given in Supplementary Data (Binding_energy_315_compounds.csv).

#### 2.1.4. Evaluation of the Synthetic Accessibility

The synthetic accessibility of the 315 compounds retained after docking-based screening was assessed. The score differentiates easy-to-synthesize compounds from those that are hard to synthesize and assigns a score to each compound. The synthetic score, SMILES, and other parameters are given in Supplementary Data (DeepSA_315_compounds.csv). The resulting CSV file provides the descriptive results, including the easy synthesis (ES) and hard synthesis (HS) scores for each compound. These scores range from 0 to 1, with values closer to one indicating an ES compound. Additionally, parameters such as HA_num (hydrogen acceptor number), Ring_num, and the rule of five are included for a more in-depth evaluation. The DeepSA results were filtered with an ES ≥ 0.99 threshold, yielding 105 compounds that met this cutoff and were predicted to be readily synthesizable. The sorted compounds were further analyzed.

#### 2.1.5. Pharmacophore-Based Network Analysis Using PharmacoNet

Following synthetic accessibility filtering, the shortlisted compounds were further evaluated using PharmacoNet [50] to analyze their pharmacophore interaction patterns and target-specific network relevance. The analysis was performed on 105 compounds, along with the co-crystal ligand; the fit scores of all compounds were calculated and compared with the co-crystal ligand as a reference. The PharmacoNet score with SMILES of 105 and A1LVI is given in the Supplementary File (PharmacoNet_score_105_molecules.csv). The co-crystal ligand yielded a fit score of 46, which was used as a screening threshold (≥46), resulting in the retention of 32 compounds that met this criterion.

#### 2.1.6. Structural Diversity Assessment and Synthetic Feasibility

**Figure 4:**
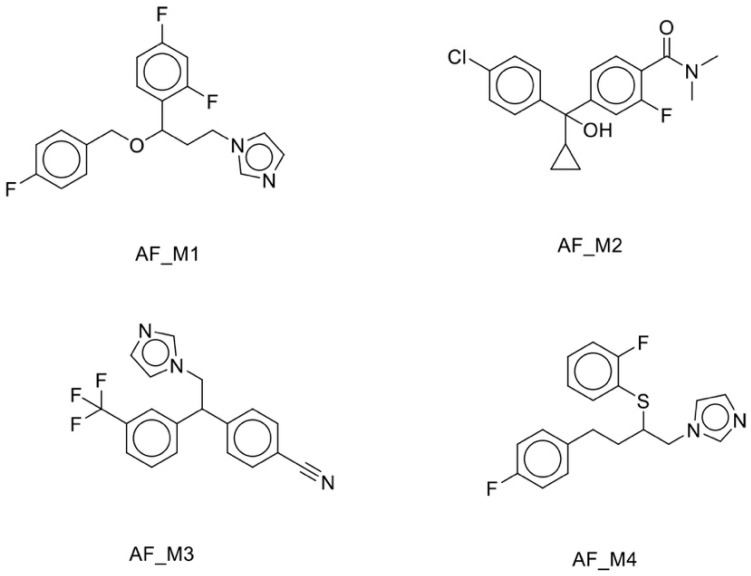
Two-dimensional representation of the compounds AF_M1–AF_M4.

Following PharmacoNet screening, the 32 retained compounds were subjected to 2D structural visualization to assess scaffold diversity and the distribution of key functional groups. The 2D representation of the molecules is given in Supplementary Data (Two-dimensional_32_molecule.pdf). Particular importance was given to compounds demonstrating favourable binding interactions and distinct chemical attributes. Based on structural diversity and interaction profiles, four top-ranking compounds, such as A49, A2813, A2384, and A3307 (in Two-dimensional_32_molecule.pdf), were selected as potential antifungal GWT1 inhibitors, along with the co-crystal ligand as a reference compound. For simplicity, from now on, the final top molecules A49, A2813, A2384, and A3307 will be referred to as AF_M1, AF_M2, AF_M3, and AF_M4, respectively. The 2D structures of the selected compounds are given in Figure 4. It is important to discuss the synthetic accessibility of the four hits. Although each of them contains a stereogenic center, none is expected to pose a significant synthetic challenge. The benzylic stereocenters in AF_M1, and AF_M3 can be accessed via standard C–C bond-forming steps such as benzylic alkylation, imidazole epoxide opening, or 1,1-diaryl-type assembly. The above arguments are well documented in the imidazole antifungal literature [51]. The tertiary alcohol stereocenter in AF_M2 can be reliably installed by nucleophilic addition of an aryl- or cyclopropyl-organometallic reagent to the corresponding ketone, and it is also well studied [52]. In the case of AF_M4, the key challenge might be establishing the C–S stereocenter and selectively alkylating the imidazole at N1, which can be addressed by first opening a chiral 4-(4-fluorophenyl)butane-derived epoxide with imidazole, followed by reaction with 2-fluorothiophenol, giving the target in high enantiomeric purity [53]. In each case, the compound can be obtained as a racemate using well-established chemistry, without recourse to asymmetric catalysis or chiral resolution [54].

The top final selected novel molecules, AF_M1–AF_M4, are fluorinated molecules featuring extended aromatic systems linked through ether, thioether, methylene, or cyclopropyl groups to nitrogen heterocycles or polar groups, closely resembling the pharmacophoric features of the clinical candidate manogepix, which fits into the palmitoyl-CoA binding pocket. AF_M3 shows the greatest potential due to its trifluoromethyl group, benzonitrile structure, which provides improved lipophilicity and hydrogen-bond acceptor properties, enabling strong engagement with the deep hydrophobic cavity. AF_M1 and AF_M4 also exhibit extended structures with multiple fluorine atoms that support important van der Waals interactions. Conversely, the higher polarity of AF_M2, caused by its dimethylamide groups may reduce binding efficiency and membrane permeability. The binding energy, PharmacoNet score, physicochemical, drug likeness, pharmacokinetic, toxicity parameters, and binding interacting amino acids of final AF_M1–AF_M4 are given in Table 1.

### 2.2. Binding Interactions Analysis

**Figure 5:**
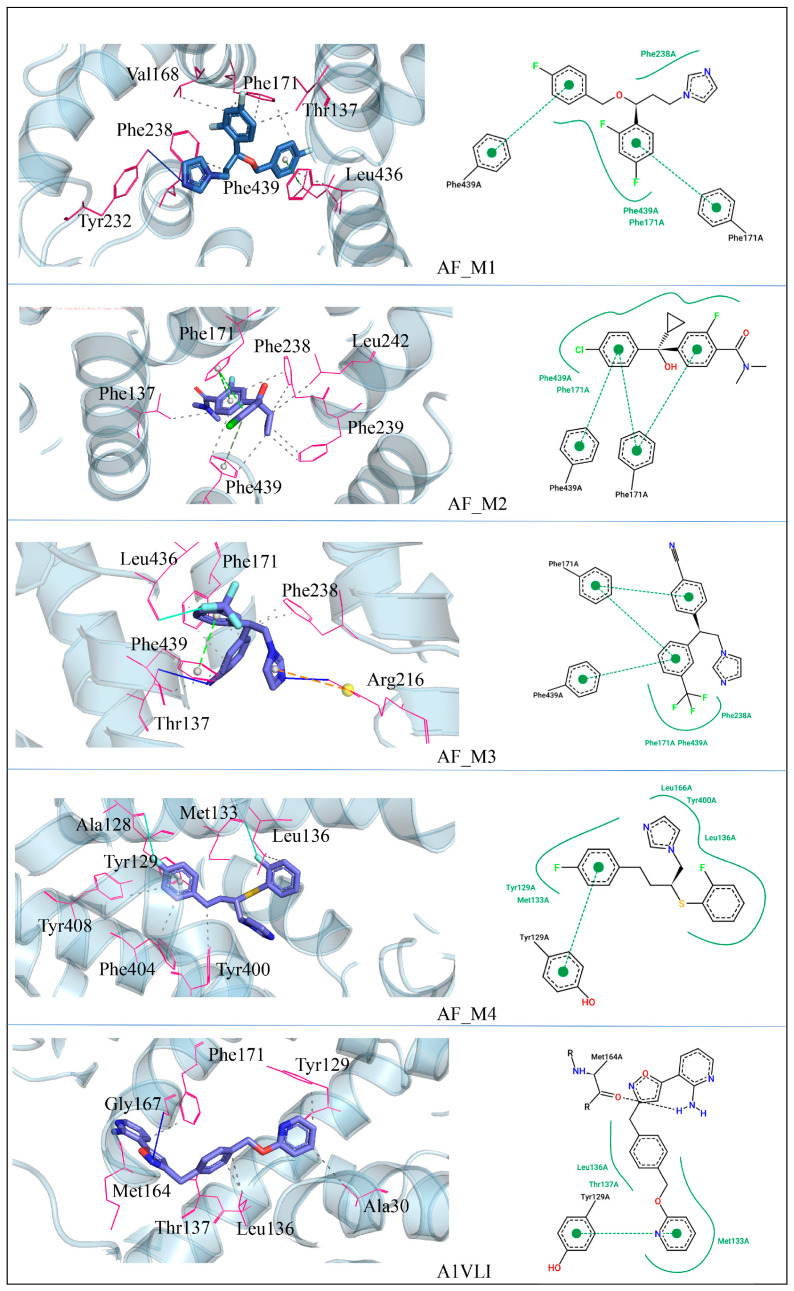
Protein–ligand interaction maps for AF_M1–AF_M4 and the co-crystal ligand A1LVI docked into GWT1.

The binding energy of AF_M1, AF_M2, AF_M3, AF_M4, and A1LVI was found to be −9.86, −9.74, −9.84, −9.73, and −9.70 kcal/mol, respectively. The binding interaction analysis was performed on the selected complexes to investigate hydrogen bonding, hydrophobic contacts, and other non-covalent interactions, and the results are shown in Figure 5. As summarised in Table 1, AF_M1 and AF_M2 demonstrated stable hydrogen bonding interactions, particularly involving residues such as Tyr232, Thr137, and Arg216, along with multiple hydrophobic contacts with Phe171, Phe238, and Phe439. Notably, π–π stacking interactions with Phe439 were consistently observed, indicating strong aromatic stabilization within the active site. Although AF_M3 and AF_M4 did not form any conventional hydrogen bonds, they showed extensive hydrophobic interactions and π-stacking contacts, particularly with phenylalanine and tyrosine residues lining the binding pocket. AF_M4 also displayed halogen-bonding interactions, which may contribute to enhanced binding stability. The co-crystal ligand A1LVI formed hydrogen bonds with Met164 and Gly167, along with several hydrophobic interactions involving Leu136, Thr137, and Phe171. Comparative analysis revealed that the selected compounds conserved key hydrophobic and aromatic interaction patterns observed in the co-crystal ligand, suggesting effective mimicry of essential binding determinants required for GWT1 inhibition. Overall, the analysis revealed conservation of key binding interactions, suggesting that the selected candidates effectively mimic essential pharmacophoric features required for GWT1 inhibition and supporting their potential as promising antifungal GWT1 candidates.

### 2.3. MD Simulation and Binding Free Energy Analysis of Selected GWT1 Inhibitors

**Figure 6:**
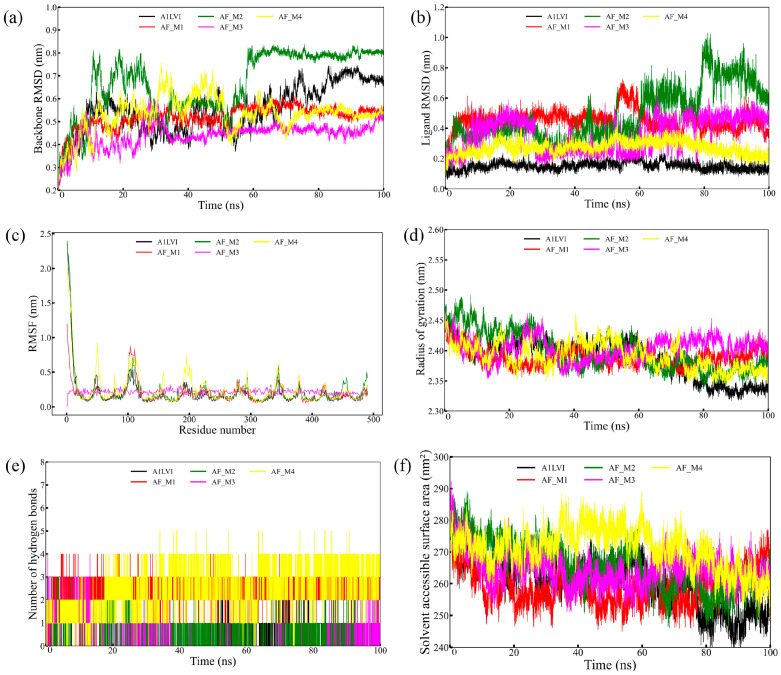
Statistical parameters from the MD simulation. (**a**) GWT1 backbone RMSD, (**b**) Ligand RMSD, (**c**) RMSF, (**d**) Radius of gyration, (**e**) Number of intermolecular hydrogen bonds, and (**f**) solvent accessible surface area.

In order to explore the dynamic stability of the selected molecules in complex with GWT1, a 100 ns MD simulation was performed. Several statistical metrics, including protein backbone RMSD (Figure 6a), ligand RMSD (Figure 6b), root-mean-square fluctuation (RMSF) (Figure 6c), radius of gyration (RoG) (Figure 6d), intermolecular hydrogen bond analysis (Figure 6e), and solvent accessible surface area (SASA) (Figure 6f), were analyzed. The average, maximum, and minimum protein backbone RMSD, ligand RMSD, RMSF, and RoG values of each complex are calculated and given in Table 2.

#### 2.3.1. Protein Backbone RMSD

To evaluate the overall conformational and binding stability of the protein–ligand complex, the protein backbone RMSD was calculated from the MD simulation trajectory and given in Figure 6a. A higher RMSD value indicates unfolding, while a lower value suggests greater compactness. All systems showed an initial rise in RMSD during the first 10–20 ns. The GWT1 protein backbone RMSD bound with AF_M3 showed the lowest and most stable RMSD throughout the simulation, indicating the high stability with minimal deviations. Similarly, the GWT1 backbone in complex with AF_M1 and AF_M4 stabilized after ~20 ns and remained in the range of 0.5–0.6 nm, suggesting stable trajectories. In contrast, the GWT1 backbone bound to AF_M2 exhibited greater deviation between 0–30 ns and again after 60 ns, with RMSD values reaching approximately 0.8 nm. This greater deviation suggests more pronounced conformational changes in the protein backbone. On the other hand, the GWT1 backbone bound to A1LVI showed a moderate deviation within the normal range. From a comparative perspective, backbone bound to AF_M1 and AF_M3 demonstrated comparable RMSD with the backbone bound to the co-crystal ligand, suggesting their strong potential to maintain the structural integrity of the GWT1 active site. Overall, the backbone RMSD profiles suggest that AF_M1, AF_M3, and AF_M4 maintained stable GWT1 conformations comparable to the reference complex. The average, maximum, and minimum protein backbone RMSD values for each of the studied complexes are given in Table 2, while no major variations can be observed.

#### 2.3.2. Ligand RMSD

Ligand stability within the binding pocket was assessed by monitoring deviations from the initial (native) conformation during the MD simulation. For this purpose, the RMSD of each ligand bound to GWT1 was calculated and is presented in Figure 6b. The average, maximum, and minimum RMSD values for the studied ligands are summarized in Table 2. The co-crystal ligand displayed the lowest RMSD values compared with the other small molecules bound to the GWT1 protein. Among the selected GWT1 inhibitors, compound AF_M4 exhibited highly stable behaviour, with RMSD values consistently ranging between 0.2 and 0.3 nm throughout the simulation. AF_M3 showed similarly stable and low deviations. In contrast, AF_M1 displayed a noticeable increase in RMSD around 60 ns, followed by stabilization at higher values. AF_M2 showed the largest deviation, with RMSD values rising to approximately 1.0 nm toward the end of the simulation, indicating greater mobility within the binding pocket and possibly frequent conformational rearrangements. Overall, most ligands exhibited relatively stable RMSD profiles with only minor fluctuations, suggesting they remained well accommodated within the active site of the GWT1 protein throughout the simulation.

#### 2.3.3. Root-Mean Square Fluctuation

The RMSF analysis was performed to assess the contribution of individual amino acid residues to the stability of the protein–ligand complexes and to evaluate the fluctuation of each amino acid during the MD simulation. RMSF values for each residue in the GWT1 protein are shown in Figure 6c for the complexes with AF_M1, AF_M2, AF_M3, AF_M4, and the co-crystal ligand A1LVI. No trajectory exhibited unusually high fluctuations. Across all systems, RMSF values remained below 0.6 nm for most residues, indicating stable ligand accommodation within the active site throughout the simulation. Among the complexes, the AF_M3–GWT1 system exhibited the lowest RMSF profile, with minimal fluctuations in most residues compared with the other ligands. This suggests that AF_M3 induced the least conformational changes in the protein during the simulation. The RMSF results demonstrate that the GWT1 protein maintained consistent structural stability when bound to the studied ligands, with only limited local flexibility throughout the MD trajectories.

#### 2.3.4. Radius of Gyration

The RoG provides a measure of the overall compactness and structural rigidity of the protein–ligand complex during the MD simulation. RoG values for GWT1 bound to the top selected ligands (AF_M1, AF_M2, AF_M3, AF_M4) and the co-crystal ligand (A1LVI) are presented in Figure 6d. To facilitate comparison, the average, maximum, and minimum RoG values for each complex are summarized in Table 2. The difference between the maximum and minimum RoG values serves as an indicator of conformational flexibility. For the GWT1 complexes with AF_M1, AF_M2, AF_M3, AF_M4, and A1LVI, these differences were 0.11 nm, 0.15 nm, 0.13 nm, 0.13 nm, and 0.15 nm, respectively. Visual inspection of the RoG trajectories revealed no major fluctuations across the 100 ns simulation period. All complexes exhibited stable RoG profiles, confirming the structural compactness of the systems. Among the tested ligands, the complexes with AF_M3 and AF_M1 displayed the most consistent RoG values throughout the simulation. In contrast, the GWT1–AF_M2 complex showed a slightly higher initial deviation but stabilized during the later stages. Overall, the consistently low RoG values across all complexes indicate that the GWT1 protein maintained a compact, rigid conformation when bound to the ligands studied during the MD simulation.

#### 2.3.5. Intermolecular Hydrogen Bond Interaction

Hydrogen-bond interactions between the GWT1 protein and the selected ligands are a key determinant of binding affinity and complex stability during MD simulations. Ligands that maintain a higher number of persistent hydrogen bonds generally exhibit stronger and more specific interactions with the protein, whereas fewer hydrogen bonds suggest greater reliance on other non-covalent forces. The number of intermolecular hydrogen bonds formed in each simulation system is presented in Figure 6e. Across most trajectories, the ligands exhibited relatively consistent hydrogen-bond profiles throughout the simulation. In some frames where hydrogen bonds were minimal or absent, the ligands remained inside the active site primarily through hydrophobic contacts, van der Waals interactions, and other nonpolar forces. Among the selected compounds, AF_M4 formed the most stable and abundant hydrogen bonds, typically ranging from 3 to 4, and occasionally reaching 5 throughout the simulation. This indicates robust and persistent polar interactions with key residues in the binding pocket. AF_M3 exhibited a moderate number of hydrogen bonds, consistently up to 3. In contrast, the remaining ligands (AF_M1, AF_M2, and the co-crystal ligand) displayed comparatively fewer hydrogen bonds, generally not exceeding 2, suggesting that their binding stability depends more heavily on complementary non-hydrogen-bond interactions. These results indicate that AF_M4 forms the strongest hydrogen-bond network with GWT1, contributing significantly to the favourable binding behaviour observed in the MD trajectories.

#### 2.3.6. Solvent-Accessible Surface Area

The SASA was calculated to evaluate the degree of protein surface exposure to the surrounding solvent and to assess overall compactness and conformational dynamics of the GWT1 protein in complex with the selected ligands. SASA profiles for all five complexes (GWT1 bound to AF_M1, AF_M2, AF_M3, AF_M4, and the co-crystal ligand A1LVI) are depicted in the in Figure 6f. Throughout the 100 ns MD simulation, all complexes exhibited SASA values ranging between approximately 250 and 290 nm^2^, with relatively small fluctuations, indicating that the overall fold and structural integrity of GWT1 were well preserved in the presence of each ligand. Notably, the GWT1–AF_M1 and GWT1–A1LVI complexes displayed the lowest average SASA values (259.08 nm^2^ and 260.68 nm^2^, respectively), consistent with a more compact protein conformation and reduced solvent-exposed surface. In contrast, the GWT1–AF_M4 complex showed the highest average SASA (270.93 nm^2^), suggesting a modest increase in structural flexibility and greater solvent accessibility, potentially due to subtle ligand-induced conformational adjustments in the binding pocket or surrounding regions. The complexes with AF_M2 and AF_M3 exhibited intermediate SASA values (265.49 nm^2^ and 264.45 nm^2^, respectively), reflecting a balanced degree of compactness and flexibility. Collectively, the relatively narrow range of SASA fluctuations across all systems confirms the structural stability of GWT1 when bound to the studied ligands. The observed differences in average SASA values highlight ligand-specific effects on protein conformational dynamics and solvent exposure, with AF_M4 inducing the most pronounced increase in accessible surface area while still maintaining overall complex integrity.

#### 2.3.7. Principal Component Analysis

**Figure 7:**
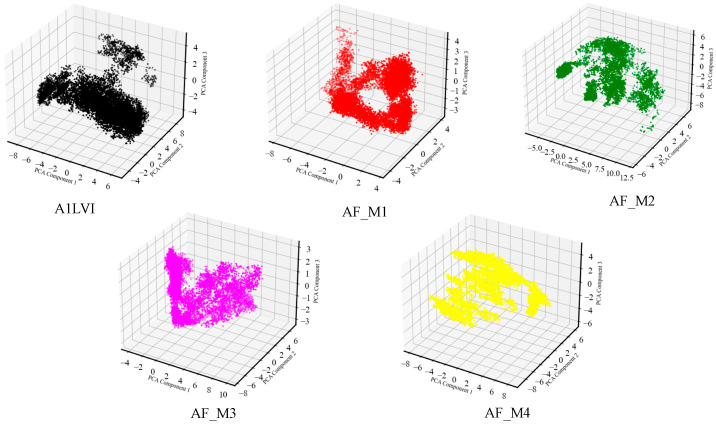
The principal component analysis of the selected molecules and cocrystal ligand A1LVI of the GWT1 protein.

Principal component analysis (PCA) was conducted on the MD trajectories to identify the dominant modes of motion and to visualize the conformational sampling of the GWT1 protein in complex with the selected ligands and A1LVI. The resulting PCA plots, projected onto the first two principal components (PC1 and PC2), are presented in Figure 7. The scatter plots reveal distinct clustering behaviours among the different ligand-bound systems. The complexes with A1LVI, AF_M1, and AF_M3 occupy compact, well-defined regions in conformational space, forming tight clusters with limited spread along both PC1 and PC2. This pattern indicates restricted conformational flexibility and stable binding poses throughout the simulation. In contrast, the complexes with AF_M2 and AF_M4 exhibit more dispersed distributions, with a broader scatter of points across the PC1–PC2 plane. This greater spread reflects increased conformational sampling and higher molecular flexibility of the protein–ligand system, suggesting that these ligands permit or induce a wider range of accessible conformations during the MD simulation. These PCA results show that while A1LVI, AF_M1, and AF_M3 encourage more rigid and consistent protein conformations, AF_M2 and AF_M4 enable greater dynamic flexibility. Such differences in conformational flexibility might affect binding kinetics, target specificity, and overall inhibitory strength. The observed tight clustering of several candidates supports their potential as stable GWT1 inhibitors, while the increased flexibility observed in others could be beneficial for adapting to pocket variations or for increasing residence time in dynamic environments.

#### 2.3.8. Free Energy Landscape

**Figure 8:**
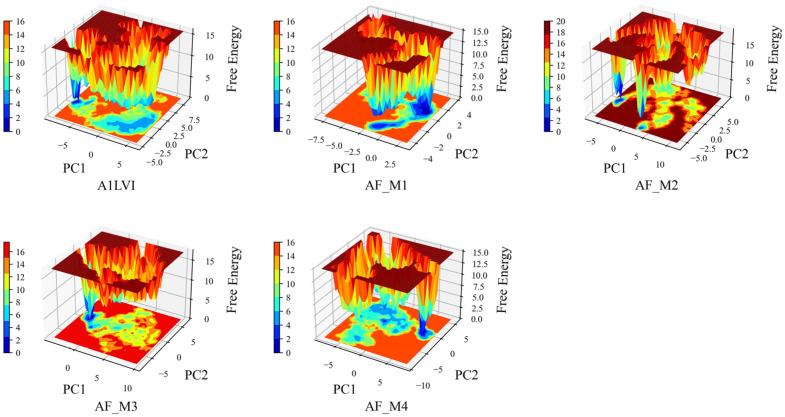
Free energy landscape of the proposed GWT1 inhibitors.

Free energy landscape (FEL) analysis, based on the first two principal components (PC1 and PC2) from PCA, was used to map the conformational stability and thermodynamic favourability of the GWT1–ligand complexes during the MD simulation. The FEL plots are shown in Figure 8, where color gradients represent Gibbs free energy with lower-energy states in blue (deep valleys) and higher-energy states in red (shallower regions). GWT1 bound to A1LVI exhibits a single, deep, and well-defined global energy minimum, reflecting high conformational stability and a strongly preferred binding pose with minimal exploration of alternative states. Similarly, the complexes with AF_M1 and AF_M3 show compact, deep energy basins at low free-energy levels, suggesting that these ligands maintain the protein in a stable, low-energy conformation during the simulation, similar to the native co-crystal ligand. The FEL for GWT1-AF_M2 complex shows a broader, more dispersed energy landscape featuring multiple shallow minima and wider sampling along both PC axes. This pattern signifies greater conformational flexibility, with the system exploring a larger number of accessible states and lower barriers between them, consistent with the previously observed dispersed PCA clustering. AF_M4 complex with GWT1 exhibits an intermediate profile with a reasonably well-defined primary energy basin, but with somewhat broader contours and a slightly shallower depth than GWT1-A1LVI, GWT1-AF_M1, and GWT1-AF_M3, suggesting moderate flexibility while still maintaining a dominant stable conformation. Together, the FEL results support the PCA findings and show that GWT1 binding to AF_M1 and AF_M3 provides thermodynamic stability similar to that of the co-crystal ligand, as indicated by deep, localized energy minima. These ligands likely promote tighter binding and a reduced entropic penalty, supporting their prioritization as promising computational candidates.

#### 2.3.9. Binding Free Energy Through the MM-GBSA Approach

The binding free energy of the AF_M1, AF_M2, AF_M3, AF_M4, and the co-crystal ligand A1LVI towards the GWT1 protein was estimated using the MM-GBSA method. From each 100 ns trajectory (10,000 frames), 2000 evenly spaced frames were extracted for MM-GBSA analysis. The calculated average binding free energy values, along with their standard deviations, are given in Table 3. More negative values indicate stronger binding affinity and greater thermodynamic favourability of the molecule towards GWT1. The average binding free energy was found to be −28.34, −25.83, −23.91, −36.64, and −36.14 kcal/mol for AF_M1, AF_M2, AF_M3, AF_M4, and A1LVI, respectively. Notably, AF_M4 exhibited the most favourable binding free energy, closely followed by A1LVI and then AF_M1. These results suggest that AF_M4 and AF_M1 showed strong affinity towards GWT1. In comparison, AF_M2 and AF_M3 showed moderately less favourable binding energies, yet still within a range indicative of significant affinity. Overall, the four proposed hit compounds exhibited substantial binding free energies, ranging from −23.91 to −36.64 kcal/mol, confirming their strong noncovalent interactions with the target protein. These MM-GBSA results support favourable predicted binding energetics, particularly for AF_M4 and AF_M1, and further justify their prioritization for experimental testing.

#### 2.3.10. Per-Residue Energy Decomposition

Per-residue energy decomposition analysis of the MD simulations revealed the specific energetic contributions of active-site residues to the binding of the designed modulators and the reference A1LVI in GWT1. The per-residue energy decomposition for all interacting amino acids is provided in the Supplementary Data (Decomposition_energy.jpg). Arg216 provided strong, favourable energies of −55.41 kcal/mol for AF_M1, −52.87 kcal/mol for AF_M2, and −58.55 kcal/mol for AF_M3, consistent with the clear polar contact shown in the AF_M3 docking pose. Phe439 consistently delivered strong, favorable contributions across AF_M1 with −29.78 kcal/mol, AF_M2 with −29.43 kcal/mol, AF_M3 with −31.73 kcal/mol, and A1LVI with −28.88 kcal/mol, matching the aromatic stacking interactions observed for this residue in multiple docking analyses. Tyr232 and Phe239 also supplied substantial favourable energies, especially for AF_M1 with−21.62 and −20.14 kcal/mol, respectively, and AF_M2 of −21.63 and −17.21 kcal/mol, corresponding, as well as for A1LVI −18.09 and −11.01 kcal/mol, respectively, consistent with the hydrophobic contacts depicted in their binding modes. Met133 contributed steady, favourable energies of approximately −10.3 to −11.9 kcal/mol across all five ligands. AF_M4 showed favourable contributions from Tyr400 (−12.08 kcal/mol), Tyr408 (−12.24 kcal/mol), Ala30 (−11.49 kcal/mol), and Leu136 (−4.76 kcal/mol), consistent with its distinct interaction network in the docking study. Val168 and Met164 showed favourable energies in most complexes (−15.5 to −18.9 kcal/mol), while unfavourable positive energies appeared for Phe238 (up to +7.85 kcal/mol), Leu242 (approximately +5 kcal/mol), Gly167 (mostly +2 to +4.5 kcal/mol), and, in AF_M4 and A1LVI, Tyr129 (+12.48 and +13.2 kcal/mol) and Phe404 (+8.24 and +14.24 kcal/mol), despite their proximity in the docking poses. Hence, the designed molecules AF_M1, AF_M2, and AF_M3 exhibited comparable or stronger favourable energetic contributions from the shared key residues than the reference molecule A1LVI, reinforcing the persistent ligand–protein contacts observed throughout the MD simulations.

### 2.4. Density Functional Theory

DFT calculations were performed to gain deeper insights into the electronic structure, chemical reactivity, and intermolecular interaction potential of the selected final compounds.

#### HOMO-LUMO Energies

**Figure 9:**
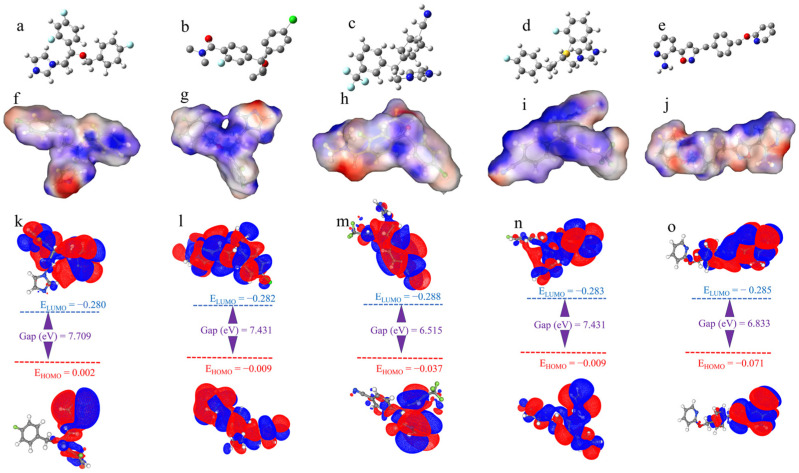
Molecular optimized geometries, electrostatic map, and molecular orbitals for the selected GWT1 inhibitors, i.e., (**a**–**o**) AF_M1, AF_M2, AF_M3, AF_M4, and A1LVI, respectively. The optimized structures are labelled with an atom numbering scheme, the electrostatic map shows the electron distribution around the respective molecules, and the molecular orbitals map is differentiated into HOMO and LUMO, along with the energy gap.

The frontier molecular orbital (FMO) energies, specifically the highest occupied molecular orbital (HOMO) and lowest unoccupied molecular orbital (LUMO), were calculated using DFT to evaluate the electronic properties, chemical reactivity, and potential for charge transfer in the selected compounds (AF_M1–AF_M4) and the A1LVI, and are given in Figure 9. The HOMO energy reflects the molecule’s ability to donate electrons, while the LUMO energy indicates its capacity to accept electrons. The HOMO–LUMO energy gap (ΔE) serves as a key indicator of overall reactivity, polarizability, and electronic stability. Smaller gaps are associated with higher reactivity, increased charge-transfer potential, and a greater likelihood of favourable intermolecular interactions. The calculated HOMO and LUMO energies, along with the corresponding energy gaps (in eV), are shown in Table 4. HOMO energies ranged from −0.258 to −0.288 Hartree, and LUMO energies from −0.009 to 0.002 Hartree, highlighting variations in electron-donating and electron-accepting tendencies among the compounds. AF_M3 exhibited the smallest HOMO–LUMO gap (6.515 eV), indicating the highest chemical reactivity, increased polarizability, and greatest electronic flexibility. This narrow gap suggests a strong tendency for charge transfer and efficient orbital overlap with complementary sites in the GWT1 binding pocket, potentially contributing to enhanced binding affinity and interaction specificity. A1LVI showed a similarly narrow gap (6.833 eV), reflecting a balanced electronic profile with moderate reactivity and good stability. In contrast, AF_M1 (7.709 eV), AF_M2 (7.431 eV), and AF_M4 (7.421 eV) exhibited larger energy gaps, indicating greater electronic stability, lower polarizability, and reduced reactivity. These wider gaps imply more rigid electronic structures, which could result in less dynamic charge redistribution and potentially weaker or more geometrically constrained interactions within the active site.

Optimized molecular geometries, electrostatic potential maps (highlighting charge distribution), and spatial distributions of HOMO and LUMO orbitals are shown in Figure 9 for each compound. These diagrams reveal localized HOMO density mainly on electron-rich aromatic or heteroatomic regions and LUMO density on electron-deficient parts, consistent with typical donor–acceptor patterns in bioactive small molecules. Therefore, the FMO analysis shows that AF_M3 and A1LVI possess the most favourable electronic properties, higher reactivity, and charge-transfer ability, thereby enabling effective molecular recognition and stable binding to GWT1. While AF_M1, AF_M2, and AF_M4 display greater electronic stability, their lower reactivity may limit optimal orbital interactions, making AF_M3 especially promising for inhibitor design owing to its electronic complementarity.

## 3. Discussion

The present study employed an integrated computational pipeline that combined AI-driven *de novo* design, molecular docking, ADMET profiling, MD simulations, binding free-energy calculations, and DFT analysis to prioritize four promising GWT1 candidates (AF_M1–AF_M4) as promising antifungal agents.

Molecular docking findings indicated that the binding energies of AF_M1 to AF_M4 ranged from −9.73 to −9.86 kcal/mol. These values were slightly more favourable than those of the co-crystal ligand A1LVI, which was at −9.70 kcal/mol, suggesting a modestly stronger predicted affinity for the GWT1 active site. The compounds share structural similarities with the clinical candidates manogepix, featuring fluorinated aromatic systems linked to nitrogen heterocycles that effectively occupy the palmitoyl-CoA binding pocket. Binding interaction analysis showed that the selected molecules formed key non-covalent contacts within the GWT1 pocket. AF_M1 and AF_M2 formed hydrogen bonds with residues such as Tyr232, Thr137, and Arg216, while all four compounds exhibited extensive hydrophobic interactions with Thr137, Phe171, Phe238, Phe439, and related amino acids. π–π stacking interactions, especially with Phe439 and Phe171, provided additional stabilization. AF_M3 was notable for its trifluoromethyl and pyridine N-oxide groups, which may enhance lipophilicity and cavity engagement, whereas AF_M4 showed useful halogen bonds.

The MD simulations provided insights into the dynamic stability of the GWT1 protein when in complex with the selected molecules AF_M1, AF_M2, AF_M3, and AF_M4 over 100 ns. Overall, the complexes kept reasonable structural integrity, although some variations appeared among the compounds. Protein backbone RMSD analysis showed that AF_M1, AF_M3, and AF_M4 exhibited stable trajectories with low deviations, similar to the reference ligand A1LVI. AF_M3 demonstrated the most consistent and lowest RMSD values, indicating strong conformational stability of the GWT1 backbone. In contrast, AF_M2 showed higher fluctuations, suggesting greater conformational changes in the protein. Ligand RMSD profiles indicated that AF_M4 remained highly stable within the binding pocket, with minimal deviations throughout the simulation. AF_M3 also showed good stability, whereas AF_M1 and AF_M2 exhibited larger shifts, particularly in the later stages, suggesting greater mobility. RMSF and RoG analyses confirmed limited local flexibility and maintained compactness across all systems, with AF_M3 showing the lowest fluctuations. Intermolecular hydrogen bonding analysis showed that AF_M4 formed the most stable hydrogen bonds, supporting its binding stability, while the other ligands depended more on hydrophobic interactions. SASA values remained steady, reflecting overall protein compactness. PCA and FEL analyses further showed that AF_M1 and AF_M3 occupy compact conformational spaces with deep energy minima, similar to A1LVI, indicating thermodynamic stability and limited flexibility. These findings suggest that AF_M1, AF_M3, and AF_M4 form relatively stable complexes with GWT1.

The MM-GBSA analysis provided a more detailed evaluation of binding affinity by accounting for dynamic behaviour during the MD simulations. The average binding free energies were −28.34 kcal/mol for AF_M1, −25.83 kcal/mol for AF_M2, −23.91 kcal/mol for AF_M3, −36.64 kcal/mol for AF_M4, and −36.14 kcal/mol for the reference ligand A1LVI. These values indicate a stronger affinity than the docking scores, which ranged narrowly from −9.73 to −9.86 kcal/mol for the selected compounds and −9.70 kcal/mol for A1LVI. Unlike the similar docking energies, MM-GBSA showed clearer differences, with AF_M4 having the most favorable affinity, slightly better than the co-crystal ligand, followed by AF_M1. AF_M2 and AF_M3 showed moderately weaker but still significant binding strengths. Per-residue energy decomposition critically corroborated the binding interactions profile. The results indicate that AF_M4 and AF_M1 form especially stable complexes with GWT1, strengthening their potential as promising antifungal options.

DFT analysis was performed to understand the electronic structure and reactivity of the selected compounds AF_M1, AF_M2, AF_M3, and AF_M4. The HOMO-LUMO energy gap is a key indicator of chemical reactivity and charge transfer ability. AF_M3 showed the smallest energy gap of 6.515 eV, suggesting higher reactivity, increased polarizability, and a greater capacity to interact with the GWT1 binding pocket. This value was also similar to the reference ligand A1LVI, which had a gap of 6.833 eV. In contrast, AF_M1 had the largest gap at 7.709 eV, indicating greater electronic stability and lower reactivity. Both AF_M2 and AF_M4 showed intermediate gaps of 7.431 eV. These DFT parameters indicate that AF_M3 has particularly favourable electronic properties among the candidates. The results suggest that these molecules, particularly AF_M3, may be promising GWT1 candidates for antifungal development.

Collectively, these multi-level in silico results position AF_M4 and AF_M1 as particularly strong candidates due to their superior binding energetics and dynamic stability, while AF_M3 offers favourable electronic properties. This work highlights the power of AI-assisted *de novo* design, combined with physics-based validation, to accelerate the discovery of selective GWT1 candidates, addressing the urgent need for new antifungals amid increasing resistance and limited treatment options.

### 3.1. Limitations

Despite the comprehensive multi-stage computational workflow used in this study, several limitations must be acknowledged. All findings depend solely on in silico predictions from molecular modeling, docking, MD simulations, MM-GBSA calculations, and DFT analysis. Although docking enrichment in this study was modest, the conclusions are supported by convergence across multiple computational layers rather than docking alone. No experimental validation, such as enzymatic inhibition assays, minimum inhibitory concentration (MIC) testing against fungal strains, or biophysical binding studies, was conducted. As a result, the actual antifungal activity, potency, and selectivity of the identified compounds (AF_M1–AF_M4) remain unconfirmed. The study used a single structural model of GWT1 based on the available cryo-EM template. Cross-species sequence and structural conservation, as well as the effect of known or potential resistance mutations in pathogenic fungi (such as *Candida auris* or *Aspergillus fumigatus*), were not examined. This limits the applicability of the results across different fungal species. Additionally, the ADMET profiling relied on ML predictions, which, although efficient, may have inherent uncertainties compared to experimental pharmacokinetic and toxicity data.

### 3.2. Future Directions

The final promising compounds identified in this study, especially AF_M4 and AF_M1, which demonstrated superior MM-GBSA binding free energies and dynamic stability, along with AF_M3, which exhibited favourable electronic reactivity, are suggested for experimental evaluation and validation. In vitro GWT1 enzyme inhibition assays, followed by antifungal susceptibility testing (MIC determination) against clinically relevant fungal pathogens, including multidrug-resistant strains such as *Candida auris*, should be prioritized. Direct comparison with the reference inhibitor, manogepix, will help benchmark their relative potency and spectrum. Structure–activity relationship (SAR) studies, guided by current binding poses and interaction profiles, combined with iterative AI-driven optimization, can be used to enhance affinity, selectivity, and pharmacokinetic properties. Selectivity screening against the human ortholog PIG-W is crucial to reduce off-target effects and verify fungal specificity.

## 4. Materials and Methods

In this study, an integrated computational approach combining machine learning (ML) and physics-based methods was employed to identify promising antifungal modulators of the GWT1 target. Novel chemical structures were generated via *de novo* molecular design using the reinforcement-learning-based tool REINVENT4 [55], starting from known active antifungal compounds. These generated candidates underwent molecular docking with the validated GNINA protocol [56], which uses a convolutional neural network-based scoring function for enhanced pose prediction and ranking. To further prioritize compounds, synthetic accessibility was assessed using the deep learning model DeepSA [57], and pharmacophore-based screening with PharmacoNet [50] was performed. Selected promising candidates were then subjected to physics-based MD simulations using GROMACS2023.4 [58] to evaluate conformational stability and binding dynamics. Finally, quantum chemical calculations were performed employing DFT to provide detailed electronic and energetic insights.

Many of the computational tools utilized in this workflow were accessed via the SilicoXplore platform [59], a cloud-based integrated environment for comprehensive in silico drug discovery. SilicoXplore incorporates several widely used third-party tools, including GNINA, AutoDock Vina v1.2 (ADV) [60], PLANTS [61], DiffDock [62], Metatdock [60], Modeller [63], PLIP [64], fPocket [65], DeLA-Drug [66], ADMET-AI [67], and GROMACS [68], while adhering to their original licensing terms and conditions, and the original publications must be cited. In addition, it integrates multiple proprietary ML-assisted modules, including ToxAI, SilicoCG, QSAR, MolConv, SilicoScreen, Rescoring, and PharmFrag, to support various stages of the drug design pipeline. This hybrid ML-physics framework, facilitated by the SilicoXplore platform, enabled a streamlined, multi-layered evaluation of chemical space to discover novel GWT1-targeted antifungals.

### 4.1. Antifungal Compounds Library Collection and Data Curation

To identify active antifungal compounds, two well-known molecular databases, Selleckchem (Selleck Chemicals LLC, Houston, TX, USA) [30] and ChEMBL [31], were explored. Selleckchem is a leading global provider of high-quality small-molecule inhibitors, bioactive compounds, screening libraries, antibodies, proteins, and biochemical reagents, widely used in biomedical and drug discovery research. ChEMBL, maintained by the European Bioinformatics Institute (EMBL-EBI), is a manually curated, open-access database of bioactive molecules with drug-like properties that combines chemical structures, bioactivity data, and genomic information to support drug discovery and the translation of genomic data into effective therapeutics. A thorough exploration of both databases resulted in 76 compounds selected from Selleckchem and 137 from ChEMBL. Both datasets were merged, and duplicates and chemically unstable molecules were removed. Additionally, molecules with molecular weights larger than 500 Da were removed, and finally, 174 molecules were retained. The 174 selected molecules exhibited a broad range of pharmacophoric features and high structural diversity. It is expected that *de novo* molecules generated from this dataset may exhibit crucial pharmacophoric features and significant diversity. The SMILES strings of these molecules are provided in Supplementary Data (Antifungal_compounds.csv).

### 4.2. Generation of Molecular Library Using REINVENT4

REINVENT4 [55] is a powerful tool for designing molecules and can be used for tasks such as creating new molecules from scratch (Reinvent model), modifying molecular structures, scaffold hopping (Linkinvent), R-group replacement, designing molecular libraries (Libinvent), and optimizing molecules (Mol2Mol model) [55]. The tool uses reinforcement learning, neural networks, and transfer learning to create new molecules. Prior models are stored in the “priors” folder, while configuration files for different run modes are found in “configs/toml.” The selection of the model and run mode is tailored to the specific case study. In the current study, the Mol2Mol model was used to generate molecules, implementing a beam-search decoding strategy and a sampling run mode. A SMILES file containing 174 antifungal compounds was provided as input to REINVENT4, and molecules were generated for subsequent analysis. The output was obtained as a CSV file containing the generated molecules’ SMILES and their respective Tanimoto scores.

### 4.3. In Silico Pharmacokinetic Analysis and Toxicity Assessment

In silico ADMET prediction is essential in drug discovery to evaluate the pharmacokinetic and safety profiles of potential drug candidates. It ensures compounds possess drug-like properties and are safe for human use. Early ADMET screening helps filter out unsuitable compounds, reducing the risk of failure in later stages of development. Molecules generated by REINVENT4 were analysed using the ADMET-AI tool [67] via the SilicoXplore platform. ADMET-AI utilizes Chemprop-RDKit to predict the ADMET properties of compounds [67]. In the present study, several key physicochemical and ADMET parameters were evaluated to assess the drug-likeness, potential for oral bioavailability, and safety profile of candidate small-molecule modulators targeting GWT1. Molecular weight (MW) was considered, as values typically below 500 Da favor better membrane permeability and oral absorption, in line with established guidelines such as Lipinski’s Rule of Five. Hydrophobicity, quantified by the partition coefficient (logP), was examined because an optimal range (commonly 1–5) balances sufficient lipophilicity for crossing lipid membranes with adequate aqueous solubility to prevent precipitation in the gastrointestinal tract. The number of hydrogen-bond acceptors (HBA) and hydrogen-bond donors (HBD) was included, since excessive counts (HBA >10 or HBD >5) can impair passive permeability by forming too many interactions with water, thereby reducing absorption. Total polar surface area (TPSA) was evaluated, as values generally ≤140 Å^2^ are linked to better oral bioavailability by indicating a molecule’s ability for hydrogen bonding without significantly hindering membrane permeation. The quantitative estimate of drug-likeness (QED) score was calculated to provide a composite measure of overall drug-likeness, integrating multiple physicochemical properties into a single probabilistic index that reflects how closely a compound resembles known oral drugs. For pharmacokinetic and safety considerations, predicted bioavailability and human intestinal absorption (HIA) were evaluated to gauge the fraction of the dose likely to reach systemic circulation after oral administration. Toxicity risks were addressed through predictions of Ames mutagenicity (indicating potential genotoxicity), carcinogenicity, and drug-induced liver injury (DILI), which help flag compounds with elevated safety liabilities early in the discovery process. Collectively, these parameters enabled a multifaceted filtering and prioritization of candidates, ensuring that selected modulators not only exhibit promising target engagement but also possess favourable profiles for further development as orally active antifungal agents.

### 4.4. Molecular Docking

#### 4.4.1. Selection and Preparation of the Receptor

The present study aims to develop novel GWT1 inhibitors using an ML-assisted *de novo* approach to disrupt the GPI anchor biosynthesis pathway and impair fungal cell wall integrity, ultimately inhibiting fungal growth [17]. The crystal structure of the GWT1 protein (PDB ID 8XIK) bound with the inhibitor A1LVI (Manogepix) was obtained from the RCSB Protein Data Bank [69]. A thorough evaluation of multiple criteria was considered to select the most suitable crystal structure, including experimental source organism, methodology, resolution, and amino acid mutations. The selected structure in the PDB was determined by electron microscopy, with *Saccharomyces cerevisiae* as the source organism, and a resolution of 3.55 Å. This structural representation comprises 490 amino acid residues, and no amino acid mutations were observed. The protein under investigation is in complex with a co-crystal ligand, A1LVI, located at its active site. The active site of GWT1 was constituted by the amino acid residues, which include Ala30, Tyr129, Leu136, Thr137, Phe171, Thr27, Ser170, and Phe404. The selected protein structure was then prepared using the Autodock tools v4.2.6 (ADT) [70]. The unwanted components, such as water molecules and any other heteroatoms, were removed from the crystal structure. The missing atoms were identified and repaired. The polar hydrogens and Gasteiger charges were added. Finally, the Autodock 4 (AD4) atom type was assigned, and the protein was saved in the .pdbqt format for the molecular docking study.

#### 4.4.2. Molecular Docking and Protocol Validation

Molecular docking simulations help predict how a small molecule orients itself when binding to a receptor, providing valuable insights into its binding strengths and specific molecular interactions. Molecular docking is a highly effective and widely used computational technique for virtual screening that has become the standard approach in structure-based drug discovery, enabling the screening of chemical libraries ranging from small to large. In the current study, GNINA was used within the SilicoXplore platform for molecular docking. GNINA is an open-source molecular docking tool that combines efficient conformational sampling with deep-learning scoring [56]. It uses a Monte Carlo-based stochastic optimization algorithm, adapted from ADV, to quickly explore thousands of possible ligand poses within the protein binding pocket, initially guided by Vina’s fast empirical scoring function. The final ranking of poses is done by an ensemble of 3D convolutional neural networks (CNNs) that analyze grid-based atomic densities and interaction patterns to predict pose quality more accurately than traditional methods. This hybrid approach improves the identification of native-like binding poses while maintaining practical computational costs.

It is crucial to thoroughly validate the docking protocol before using it to screen new chemical libraries. Self-docking and decoy set validations are used to assess the efficiency of the docking protocol in selecting active molecules over inactive ones during virtual screening. In the self-docking approach, the co-crystal ligand, A1LVI, was redrawn in OpenBabel [48], converted to three-dimensional (3D) coordinates, and Gasteiger charges and hydrogens were added, and it was saved as a .pdbqt file. The prepared A1LVI was docked at the active site of GWT1, where the co-crystal A1LVI was bound. By varying the parameters, including the active-site coordinate and grid size, the molecular docking was repeated, and the best pose from each iteration was superimposed on the co-crystal conformation of A1LVI, and the RMSD was recorded. It has been reported that the docking protocol with superimposed RMSD of less than 2 Å can reproduce a pose similar to the crystallographic conformation. Another level of docking protocol validation was performed using decoy set validation. In this approach, the 174 active antifungal molecules were used to generate 8400 inactive (decoy) molecules using the DeepCoy tool [49], which employs deep learning (DL) methods to create property-matched decoys. The combined active compounds and decoys were docked using a protocol finalized via self-docking. The docking results were analyzed by generating ROC precision curves and calculating accuracy, ROC AUC, and specificity. This process validated the molecular docking protocol and confirmed its reliability.

The molecules retained after pharmacokinetic assessment were considered for the molecular docking using the GNINA. The small molecules were prepared using the MolConv package in SilicoXplore, which employs a series of Python RDKit v2025.09 [71] functions to remove duplicates, ions, and conjugate molecules. The molecules were converted into 3D coordinates and saved in structural data format (sdf) for the molecular docking study. The validated docking protocol and the identified active site were used in the molecular docking study, and the molecules were screened by comparing their binding energies with A1LVI.

### 4.5. Estimation of Synthesis Accessibility of Compounds Using DeepSA

DeepSA is an advanced deep learning-based tool designed to predict the synthetic accessibility (SA) score of chemical compounds [57]. It evaluates how easily a molecule can be synthesized based on structural complexity, functional groups, and known synthetic routes [57]. The tool requires SMILES as the molecular input and, based on that, converts them into numerical descriptors using deep-learning models to generate a score indicating the ease of synthesis. The compounds retained after molecular docking were subjected to DeepSA analysis. It interprets molecules as ES (easy synthesis) or HS (hard synthesis) and assigns them scores accordingly. A molecule is considered easy to synthesize if its ES score is 0.5 or higher. If the ES score is below 0.5, the molecule is considered difficult to synthesize. After calculating the ES and HS of the molecules, the synthesizable molecules were considered for the next level of assessment.

### 4.6. PharmacoNet-Assisted Virtual Screening

PharmacoNet provides a deep-learning framework for modeling protein-based pharmacophores, utilizing instance segmentation to identify key protein functional groups (hotspots) and optimal pharmacophore points [50]. It evaluates ligand compatibility through a parameterized analytic function, focusing on pharmacophoric interactions rather than atomic interactions. It labels the ligand’s functional groups and aligns their poses with the generated pharmacophore model, evaluating their compatibility by assigning fit scores to the ligands. PharmacoNet was executed for the selected compounds, along with the co-crystal ligand [50]. To reduce the chemical space, the compounds’ fit scores were retrieved. A cutoff fit score based on the co-crystal ligand was applied, and only compounds with a higher pharmacophoric fit score than the co-crystal ligand were retained for further analysis. After a thorough examination and rigorous screening of the compounds, the two-dimensional (2D) structures of each retained compound were carefully examined for active functional groups and their diversity. Based on the fit score and 2D structural assessments, top molecules for GWT1 were selected.

### 4.7. Molecular Dynamics Simulation

MD simulation is a computational method used to study the dynamic behavior of biomolecules, such as proteins and small molecules, over time [72]. Top selected molecules and A1LVI bound with GWT1 were considered for a 100 ns MD simulation study. For the above complexes, an all-atom MD simulation was performed using the GROMACS2023.4 software package [68,73]. In order to generate the ligand topology, the freely available online SwissParam tool was used [74], and the protein topology files were generated using the CHARMM36 force field [75]. The MD simulation was performed with a 2-femtoseconds time step, maintaining 1 atm pressure and 300 K temperature. The system was solvated using the TIP3P [76] water model in a cubic simulation box. The system was also neutralized by adding the required amount of Na^+^/Cl^−^ ions, followed by energy minimization using the Steepest Descent algorithm. Equilibration was conducted in two ensembles, such as NVT (number of atoms, volume, temperature) followed by NPT (number of atoms, pressure, and temperature), for 5 ns each. The post-simulation analysis included protein backbone and ligand RMSD, RMSF, RoG, hydrogen-bond interactions derived, and SASA from the MD simulation trajectories. Additionally, PCA and FEL analyses were performed to evaluate conformational dynamics. From the entire set of MD simulation trajectories, 2000 protein-ligand complexes were extracted at 5 intervals, and binding free energy was calculated using the molecular mechanics generalized born surface area (MM-GBSA) method via the gmx_MMPBSA [77] package. The per-residue energy decomposition for active-site amino acids was calculated from the MD simulation trajectories.

### 4.8. Density Functional Theory Calculations

DFT is a powerful and widely used quantum-mechanical framework for studying the electronic structure and properties of atoms, molecules, and materials [78]. Unlike traditional wavefunction-based methods, DFT mainly depends on the electron density, a 3D spatial distribution of electrons, rather than the many-electron wavefunction, as established by the Hohenberg–Kohn theorems. This reformulation enables efficient calculation of ground-state properties with good scaling, making it feasible for large systems. The top-selected molecules and A1LVI were considered for DFT calculations to investigate their electronic and structural properties. All quantum chemical calculations were performed using Psi4 [79] and ORCA [80] within the SilicoXplore platform. Geometry optimization was performed using the B3LYP functional in combination with the 6-31G(d,p) basis set. The HOMO and LUMO energies were recorded. Afterwards, the calculated energies of HOMO and LUMO were used to determine various quantum chemical parameters such as energy gap, electronegativity (v), electron affinity (A), ionization energy (I), chemical potential (m), chemical softness (S), chemical hardness (g), and electrophilicity index (x) [81]. Additionally, to access detailed information on nucleophilic and electrophilic sites, a molecular electrostatic potential (MEP) calculation was performed using the Mulliken charge method.

## 5. Conclusions

An integrated, AI-driven, simulation-based computational framework was used to identify new inhibitors targeting the GWT1 enzyme, an essential part of the fungal GPI-anchor biosynthesis pathway. Starting from curated antifungal datasets, large-scale *de novo* molecule generation was performed, followed by rigorous ADMET filtering, docking validation, pharmacophore-based screening, synthetic accessibility assessment, MD simulations, MM-GBSA binding free energy calculations, and DFT analyses. This process enabled the systematic prioritization of promising candidates. Among the shortlisted molecules, AF_M1, AF_M2, AF_M3, and AF_M4 demonstrated favourable pharmacokinetic properties, strong docking scores, conserved pharmacophoric interactions, and stable dynamic behaviour within the GWT1 binding pocket. MD simulation results showed stable backbone RMSD, acceptable ligand stability, compact protein conformations, and consistent intermolecular interactions over the 100 ns trajectory. MM-GBSA calculations further supported the binding potential of these compounds, with AF_M4 showing the most favourable binding free energy (−36.64 kcal/mol), comparable to A1LVI (−36.14 kcal/mol). Additionally, DFT analysis provided insights into the electronic properties and reactivity profiles of the lead molecules, supporting their prioritization as candidate GWT1 modulators for further validation. Therefore, this study demonstrates that AI-assisted *de novo* design, combined with multi-level computational validation, can effectively accelerate antifungal drug discovery. The identified compounds serve as promising lead scaffolds for further optimization and experimental testing.

## Figures and Tables

**Table 1:** Binding energy, physicochemical properties, pharmacokinetic parameters, toxicity, and binding interaction profiles of selected compounds, along with co-crystal ligand A1LVI.

	AF_M1	AF_M2	AF_M3	AF_M4	A1LVI
Binding energy (kcal/mol)	−9.86	−9.74	−9.84	−9.73	−9.70
PharmacoNet fit score	55.47	52.03	87.46	56.31	46.00
Molecular Weight	346.35	347.82	341.34	344.43	358.94
Bioavailability	0.92	0.95	0.90	0.90	0.91
QED	0.62	0.91	0.69	0.56	0.52
Carcinogenicity	0.05	0.43	0.14	0.04	0.26
AMES toxicity	0.31	0.15	0.15	0.15	0.27
TPSA	27.05	40.54	41.61	17.82	87.60
DILI	0.38	0.24	0.49	0.41	0.50
Hydrogen bond	Tyr232	Thr137, Arg216	-	-	Met164, Gly167
Hydrophobic interactions	Thr137, Val168, Phe171, Phe238, Leu436, Phe439	Thr137, Phe171, Phe238, Phe439	Thr137, Phe238, Phe239, Leu242, Phe439	Leu136, Tyr400, Phe404, Tyr408	Ala30, Tyr129, Leu136, Thr137, Phe171
**π**-stacking	Phe439	Phe439	Phe171, Phe439	Tyr129	-
**π**-cation	-	Arg216	-	-	-
Halogen bonds	-	Leu436	-	Ala128, Met133	-

**Table 2:** Statistical data of Post MDS analysis of GWT1 protein and selected compound complexes.

Parameters		AF_M1	AF_M2	AF_M3	AF_M4	A1LVI
Backbone RMSD (nm)	Average	0.52	0.67	0.44	0.53	0.55
	Maximum	0.61	0.84	0.60	0.75	0.75
	Minimum	0.00	0.00	0.00	0.00	0.00
Ligand RMSD (nm)	Average	0.45	0.49	0.36	0.42	0.15
	Maximum	0.72	1.03	0.62	0.43	0.25
	Minimum	0.00	0.00	0.00	0.00	0.00
RMSF (nm)	Average	0.19	0.22	0.21	0.25	0.19
	Maximum	1.19	2.40	0.32	2.03	2.35
	Minimum	0.06	0.07	0.00	0.06	0.06
RoG (nm)	Average	2.39	2.40	2.40	2.39	2.39
	Maximum	2.47	2.49	2.48	2.47	2.47
	Minimum	2.36	2.34	2.35	2.34	2.32
SASA (nm^2^)	Average	259.08	265.49	264.45	270.93	260.68
	Maximum	284.04	289.11	292.24	289.16	291.28
	Minimum	245.02	245.88	249.68	249.04	236.70

**Table 3:** Average binding free energy and Standard deviation of selected molecules with Co-crystal A1LVI bound with GWT1 protein.

Compounds	Average Binding Free Energy (kcal/mol)	Standard Deviation (±)
AF_M1	−28.34	0.70
AF_M2	−25.83	1.58
AF_M3	−23.91	0.65
AF_M4	−36.64	1.21
A1LVI	−36.14	1.04

**Table 4:** HOMO-LUMO orbital energies and HOMO-LUMO gaps of selected GWT1 inhibitors.

Compounds	HOMO Energy (Hartree)	LUMO Energy (Hartree)	Gap (eV)
AF_M1	−0.280	0.002	7.709
AF_M2	−0.282	−0.009	7.431
AF_M3	−0.288	−0.037	6.515
AF_M4	−0.283	−0.009	7.431
A1LVI	−0.258	−0.017	6.833

## Data Availability

All data supporting the reported results are included in the manuscript and Supplementary Materials. Additional details or raw files are available at https://github.com/mahimakolpe/Antifungal-Project-.git (accessed on 30 March 2026). Access can be given upon reasonable request to the corresponding author.

## Supplementary Materials

The following supporting information can be accessed with permission at https://github.com/mahimakolpe/Antifungal-Project-.git (accessed on 30 March 2026).

## Abbreviations

The following abbreviations are used in this manuscript:

GPI	Glycosylphosphatidylinositol
GWT1	GPI-anchored wall transfer protein 1
ML	Machine learning
SMILES	Simplified Molecular Input Line Entry System
DeLA-Drug	Deep Learning Algorithm for Automated Design of Drug-like Analogues
MD	Molecular dynamics
MM-GBSA	Molecular Mechanics/Generalized Born Surface Area
DFT	Density Functional Theory
SPP	Similar property principle
ADT	AutoDock tools
ADV	Autodock vina
AD4	Autodock4
AUC	Area under the curve
ADMET	Adsorption, distribution, metabolism, excretion and toxicity
SA	Synthetic accessibility
RMSF	Root-mean-square fluctuation
RMSD	Root-mean-square deviation
RoG	Radius of gyration
SASA	Solvent accessible surface area
PCA	Principal component analysis
FEL	Free energy landscape
HOMO	Highest occupied molecular orbital
LUMO	Lowest unoccupied molecular orbital
